# Long-term survival rates of laryngeal cancer patients treated by radiation and surgery, radiation alone, and surgery alone : studied by lognormal and Kaplan-Meier survival methods

**DOI:** 10.1186/1471-2407-5-13

**Published:** 2005-01-31

**Authors:** Patricia Tai, Edward Yu, Ross Shiels, Jon Tonita

**Affiliations:** 1 Allan Blair Cancer Center, Saskatchewan Cancer Agency, University of Saskatchewan, Saskatchewan, Canada; 2 London Regional Cancer Center, University of Western Ontario, Ontario, Canada

## Abstract

**Background:**

Validation of the use of the lognormal model for predicting long-term survival rates using short-term follow-up data.

**Methods:**

907 cases of laryngeal cancer were treated from 1973–1977 by radiation and surgery (248), radiation alone (345), and surgery alone (314), in registries of Connecticut and Metropolitan Detroit of the SEER database, with known survival status up to 1999. Phase 1 of this study used the minimum chi-square test to assess the goodness of fit of the survival times of those who died with disease to a lognormal distribution. Phase 2 used the maximum likelihood method to estimate long-term survival rates using short-term follow-up data. In order to validate the lognormal model, the estimated long-term cancer-specific survival rates (CSSR) were compared with the values calculated by the Kaplan-Meier (KM) method using long-term data.

**Results:**

The 25-year CSSR were predicted to be 72%, 68% and 65% for treatments by radiation and surgery, by radiation alone, and by surgery alone respectively, using short-term follow-up data by the lognormal model. Corresponding results calculated by the KM method were: 72+/-3%, 68+/-3% and 66+/-4% respectively.

**Conclusions:**

The lognormal model was validated for the prediction of the long-term survival rates of laryngeal cancer patients treated by these different methods. The lognormal model may become a useful tool in research on outcomes.

## Background

Literature review [1] indicated that local control, laryngeal preservation, and survival rates of larynegeal cancer patients were similar after transoral laser resection, open partial laryngectomy, and radiotherapy. Open partial laryngectomy was reserved for patients with locally recurrent tumors. There are still some unanswered questions. Will radiation combined with surgery give a better result than single modality treatment alone? Will treatment results from the community centers follow published data from prestigious centers? After radiotherapy, radio-resistant cells theoretically may take some time to grow before recurrence. Short-term data may not reflect long-term local control and survival rates. We attempt to address these questions in the present study.

The lognormal distribution is defined as the distribution of a random variable whose logarithm is normally distributed. The purpose of this study is to validate the use of the lognormal model [2-4] by estimating the long-term survival from short-term follow-up data of laryngeal cancer treated by three different treatment methods: radiation and surgery, radiation alone, and surgery alone. We have previously validated the application of the lognormal model for small cell lung cancer [5], glottic laryngeal cancer [6], prostate cancer [7] and breast cancer [8].

This model may be useful for randomized clinical trials because it allows the prediction of long-term survival rates several years earlier than is possible by using the standard actuarial life table/Kaplan-Meier method of calculation [9].

The idea that long-term survival rates can be estimated from short-term follow-up data is attractive because this method shortens the delay in further research to improve cancer treatment. The validation of the lognormal model has two phases. Phase 1 tests the goodness of fit to a lognormal distribution of the survival times of those cancer patients who died with disease.

Phase 2 attempts to verify the lognormal model, which uses short-term follow-up data to predict long-term survival rates. These survival rates are then compared with values calculated by the Kaplan-Meier life table method from available long-term data. The second phase has been difficult to implement because of the general lack of large number of patients with sufficiently long follow-up information. With the SEER database [10], the validation of the lognormal model is now possible.

## Methods

We analyzed a total of 907 cases of laryngeal cancer treated by three treatment methods: radiation and surgery (248 patients), radiation alone (345 patients), and surgery alone (314 patients), registered in two registries, Connecticut and Metropolitan Detroit, from 1973–1977 with known survival status up to 1999 extracted from the SEER database. Fourty-seven patients with unknown survival time and 165 patients with missing treatment methods were excluded. A table of the patient characteristics was listed in Table 1.

The cause-specific survival time was defined as the interval from the date of diagnosis to the date of death from laryngeal cancer or the date of last follow-up for censoring purposes, if the patient was alive and still being followed at the time of analysis. Patients who died of other causes were also censored at date of death.

### Phase 1 – Test of goodness of fit for lognormality

Testing for lognormality was done separately for laryngeal cancer patients who died with disease (as distinct from those who died of an intercurrent disease) treated by: radiation and surgery, radiation alone, and surgery alone. For Phase 1, a minimum chi-square method was used to estimate the standard deviation S and mean M of the log_10 _(survival time) of the distribution of patients who died of the cancer. The minimum chi-square test was run by a Microsoft Excel program. A range of S-values and M-values were tested to reduce the chi-square values to a minimum. In order to determine if the observed survival times for a given cohort are lognormally distributed, the results are given in terms of probability levels of significance P for the chi-square estimates which correspond to a minimization of chi-square and hence a maximization of P. The test statistic of the minimum chi-square test was minimized by varying the parameters and the P-value gave the significance of the test. The class intervals were in the powers of 2 in months of the survival time, such as 0–2, >2–4, >4–8, >8–16, and so on. The number of cases in each interval should not be less than 5. The null hypothesis being tested is H_0_: that there is no difference between the observed survival times and the expected survival times calculated from a lognormal distribution with a specified S and M. If P < 0.05 the null hypothesis is rejected.

### Phase 2 – Validation of the lognormal model

A second computer program for the Phase 2 of the study was also run using Microsoft Excel to estimate the cured fraction C by using a maximum likelihood method described by Boag [4]. Using the lognormal model, the standard deviation (S) was fixed, and only the two remaining parameters, the mean (M) and the cured fraction among all patients (C), were kept floating. Multiple iterations converged to a stable solution for C. The cause-specific survival rate (CSSR) at time τ = [C + (1-C) × Q] × 100%, where Q = the integral of the lognormal distribution between the limits τ and infinity, τ = long-term survival time, C was calculated by the maximum likelihood method.

The five-year cohort extended from 1973 to the end of 1977. Prediction of the long-term CSSR were made after one year follow-up, i.e. at the end of 1978, for the laryngeal cancer patients with three different treatments: radiation and surgery, radiation alone, and surgery alone. The predicted CSSR were then validated by comparing with the results calculated by the Kaplan-Meier method using the actual follow-up data available up to 1999.

Log-rank tests were performed for the three different treatment groups over the whole time period in order to see if there was a difference in CSSR between treatments.

## Results

Using data from 1973–1977, the minimum chi-square tests verified that the survival times of the patients who died of laryngeal cancer and who were treated by three treatments followed three different lognormal distributions. At minimum chi-square, the S and M values, the numbers of patients N and the P values of the minimum chi-square tests for the three different treatments are listed in Table 2.

As in a prospective trial at interim analysis, Phase 2 was performed for each of the five-year cohort periods after one year of follow-up. Phase 2 predicted, using the maximum likelihood method, the long-term 10-, 15-, 20-, and 25-year CSSR. An S value was selected for the maximum likelihood method, so that the prediction curve obtained by the lognormal model would best fit the Kaplan-Meier graph *at one year *after the five-year cohort period. Hence at the time of prediction by the lognormal model, the long-term data were not known yet. Table 3 lists the results for each treatment arm and their comparison with calculation obtained by the Kaplan-Meier method. The predictions were validated by comparing them with the Kaplan-Meier calculation using available data up to 1999.

The 25-year CSSR were predicted by the lognormal model after only short term follow-up to be 72%, 68% and 65% for treatments by radiation and surgery, radiation alone, and surgery alone respectively. The 25-year CSSR were found to be 72+/-3% (one standard error), 68+/-3% and 66+/-4% respectively by Kaplan-Meier method. Long-term survival rates at other years, e.g. 10-, 15-, and 20-year CSSR, were all within one standard error compared with the Kaplan-Meier calculations.

There were no statistically significant differences between the CSSR of the three different treatment groups for all stages combined (p = 0.35 by log-rank test for the three treatments).

**Figure 1:**
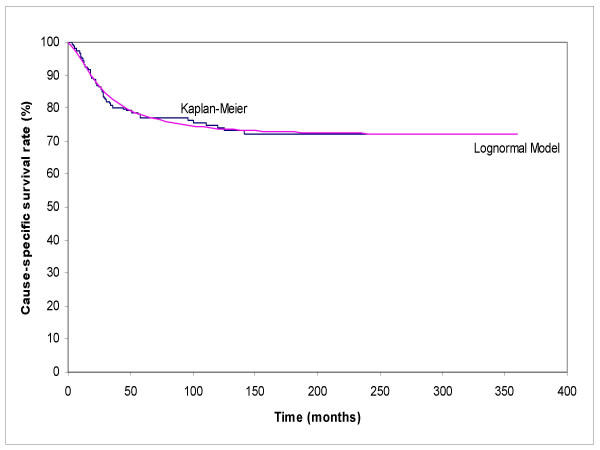
Cause-specific survival for all patients with laryngeal cancer treated by radiation and surgery estimated by the lognormal model at 1978 versus actuarial survival calculated by the Kaplan-Meier method at 1999.

**Figure 2:**
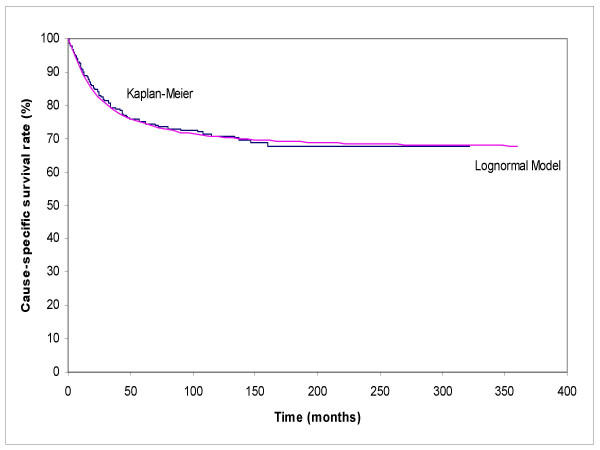
Cause-specific survival for all patients with laryngeal cancer treated by radiation alone estimated by the lognormal model at 1978 versus actuarial survival calculated by the Kaplan-Meier method at 1999.

**Figure 3:**
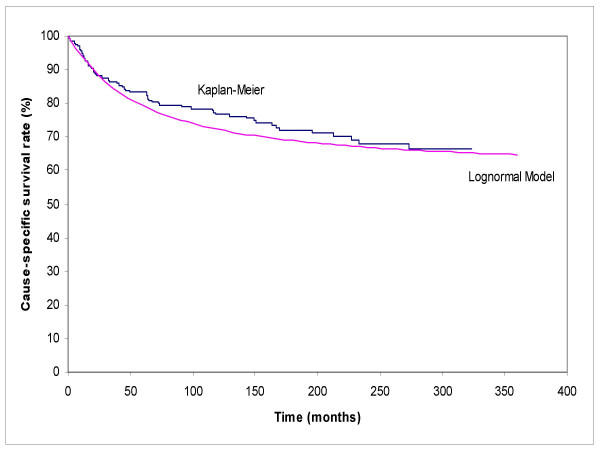
Cause-specific survival for all patients with laryngeal cancer treated by surgery alone estimated by the lognormal model at 1978 versus actuarial survival calculated by the Kaplan-Meier method at 1999.

Figures 1, 2 and 3 show the comparisons of the three different treatments: radiation and surgery, radiation alone, and surgery alone respectively for laryngeal cancer in the Kaplan-Meier graph at the year 1999 compared with the lognormal model prediction curve which could be obtained at 1978.

The SEER database provides localized and regional disease stages, instead of T1N0 or T2N0 stages, etc. The lognormal model prediction of the 25-year CSSR for 454 patients with localized stage disease were 84%, 75%, and 80% for treatments by radiation and surgery, radiation alone, and surgery alone respectively. The 25-year CSSR calculated by the Kaplan-Meier method were 83+/-4%, 75+/-4%, and 77+/-6% respectively (p = 0.08, log-rank test for the three treatments). For 286 patients with regional stage disease, the 25-year CSSR were 62%, 58%, and 52% respectively, and compared with Kaplan-Meier method were 63+/-6%, 58+/-7%, and 46+/-6% respectively (p = 0.76, log-rank test for the three treatments).

## Discussion

The current study demonstrates the application of the lognormal model to a population based study. The lognormal model is being applied in a manner that can be applied to prospective trials in practice. Our previous publication about the application of the lognormal model was for different stages of laryngeal cancer patients treated by one radiation oncologist, Dr. M. Lederman in the Royal Marsden Hospital, United Kingdom [6]. The current study shows that the lognormal model is applicable in different scenarios.

Detroit and Connecticut registries were chosen because they have the earliest data starting from 1973 and they include a large population of both white and black patients. Generally cause-specific death rates underestimate the mortality associated with a diagnosis of the specific cancer, because some patients died of other causes[11]. Gamel *et al*.[12] found that the follow-up time should be one standard deviation beyond the mean of the survival time so as to obtain stable results. In the current study, five-year cohorts extended from 1973 to the end of 1977 were used. Stable results for prediction of the long-term CSSR were obtained after one year of follow-up.

In this study, for localized stage disease 5-year CSSR were 91%, 83%, 95% for treatments by radiation and surgery, radiation alone, and surgery alone respectively with Kaplan-Meier method.

Jones *et al*.[13] found that the 5-year tumor-specific survival for those treated by radiation was 87% and for those treated by surgery was 77% (p = 0.1022). Both radiation and surgery are equally effective for treating early stage laryngeal carcinoma. In the current study, there were marginal difference in the results of the three treatments for localized stage patients.

Jorgensen *et al*.[14] found that among patients with T1 glottic carcinomas the 5-year locoregional control rate was 88%, i.e. 88% of patients were cured by radiotherapy alone. The 5-year disease-specific survival (DSS) was 99%, i.e. salvage surgery added approximately 11% to the survival of T1 glottic patients. Only 4% (12/312) of T1 glottic patients underwent laryngectomies. Locoregional control among T2 glottic patients was 67% and the DSS 88%, and 18% (41/233) of patients underwent laryngectomies. The corresponding results among T3 glottic patients were 30% and 59%, about 50% of patients underwent laryngectomies. For T3 glottic carcinomas, initial surgery did not produce better survival rates.

Franchin *et al*.[15] studied T1 and T2 glottic carcinoma. The 5-year and 10-year overall survival rates were 83% and 63.5%, respectively. The overall 10-year local control rate for patients with T1 and T2 glottic carcinoma was 89%.

In this study, the treatment results were marginally different for localized stage (p = 0.08, log-rank test for the three treatments) and the treatment results were similar for regional stage (p = 0.76, log-rank test for the three treatments). For localized stage combination radiation and surgery may have more certainty of disease control and hence long-term survival benefit. The treatment results were similar for regional stage because the patients were diagnosed late and disease control may be more difficult. The predicted survivals were within one standard error of the Kaplan-Meier estimations for both localized and regional stages. It shows that the prediction method can work for both good and poor prognosis cases.

The practical value of this study is that this lognormal model may be used for prediction of the results of prospective trials earlier than the Kaplan Meier method. This lognormal model may become a useful tool in research about outcomes. Use of this lognormal model could result in more rapid advances in cancer treatment and have the potential benefit of a reduction of cost in cancer research.

## Competing interests

The author(s) declare that they have no competing interests.

## Authors' constributions

PT: Data analysis and writing of the manuscript.

EY, RS: Critical appraisal of the manuscript.

JT: Data analysis and critical appraisal of the manuscript.

## Pre-publication history

The pre-publication history for this paper can be accessed here:



## Figures and Tables

**Table 1:** Patient Characteristics

Total number of patients	907	
Median age	62 (range 21–96)	

	Patient	Percent
Race:		

White	828	91%
Black	75	8%
Others	4	1%
Sex:		
Male	780	96%
Female	127	14%
Stage:		
Localized	454	50%
Regional	286	31%
Distant	71	8%
Unstaged	50	6%
In Situ	46	5%

**Table 2:** Phase 1 results for the three different treatments

Treatment method	S	M	N	P
Radiation and surgery	0.3623	1.3413	57	0.26
				
Radiation alone	0.5279	1.2740	86	0.66
				
Surgery alone	0.6938	1.5057	73	0.32

Abbreviations:

S = standard deviation of the lognormal distribution;

M = mean of the lognormal distribution;

N = number of patients;

P = P value for the minimum chi-square test.

**Table 3:** Cause-specific survival rates after one year of follow-up as predicted by the lognormal model compared with the actuarial results as calculated by the Kaplan-Meier method

(A)	Radiation and Surgery:					
						
		Cause-specific survival rate (%)				
Method	Year	10 y	15 y	20 y	25 y	C × 100%
Lognormal	1978	74	73	72	72	72
KM	1999	74 ± 3	72 ± 3	72 ± 3	72 ± 3	--
						
(B)	Radiation alone:					
						
		Cause-specific survival rate (%)				
Method	Year	10 y	15 y	20 y	25 y	C × 100%
Lognormal	1978	71	69	68	68	67
KM	1999	71 ± 3	68 ± 3	68 ± 3	68 ± 3	--
						
(C)	Surgery alone:					
						
		Cause-specific survival rate (%)				
Method	Year	10 y	15 y	20 y	25 y	C × 100%
Lognormal	1978	74	70	67	65	61
KM	1999	77 ± 3	72 ± 3	68 ± 3	66 ± 4	--
						

y, year;

C × 100%, cured rate estimated by the lognormal model;

KM, Kaplan-Meier method ± one standard error.
